# Hepatic Angiomyolipoma Mimicking Hepatocellular Carcinoma: Magnetic Resonance Imaging and Clinical Pathological Characteristics in 9 Cases

**DOI:** 10.1097/01.md.0000464204.63296.9b

**Published:** 2015-01-16

In the article “Hepatic Angiomyolipoma Mimicking Hepatocellular Carcinoma: Magnetic Resonance Imaging and Clinical Pathological Characteristics in 9 Cases”,^1^ which appeared in Volume 93, Issue 28 of *Medicine*, the corresponding author's name, Yong-Ping Yang, M.D., was omitted from the author list. The correct author list is given below. The article has since been corrected and reposted in the issue.

Chun-Ping Wang, M.Phil., Hong-Yan Li, M.D., Hong Wang, M.D., Xiao-Dong Guo, M.Phil., Chang-Chun Liu, M.Phil., Shu-Hong Liu, M.Phil., Xu-Dong Gao, M.Phil., Jian-Hui Qu, M.D., Ze Liu, M.D., Xiu-Juan Chang, M.Phil., Yin-Ying Lu, M.D., Zhen Zeng, M.D., Min Lou, M.Phil., and Yong-Ping Yang, M.D.
